# The Effect of Pandemic Movement Restriction Policies on Children's Physical Fitness, Activity, Screen Time, and Sleep

**DOI:** 10.3389/fpubh.2021.785679

**Published:** 2021-12-06

**Authors:** Shawnda A. Morrison, Kaja Meh, Vedrana Sember, Gregor Starc, Gregor Jurak

**Affiliations:** Faculty of Sport, University of Ljubljana, Ljubljana, Slovenia

**Keywords:** cardiorespiratory fitness, COVID-19, musculoskeletal strength, 24-hour movement behavior, environmental epidemiology

## Abstract

**Background:** The negative impact of isolation, confinement, and physical (in)activity due to pandemic movement restriction has been well-documented over the past year, but less is known on the impact of these policies on children's physical fitness. This study was designed to determine the effects of pandemic movement restriction policies on the 24-hour movement behavior (24-HMB) of children, and whether any alterations are reflected in worsening physical fitness outcomes determined via direct testing.

**Methods:** A two-phase, repeated-measures study with matched controls was conducted. Phase One: *N* = 62 schoolchildren (*N* = 31 female) completed self-assessment questionnaires on 24-HMB in October 2018 (pre-pandemic) and again in April 2020, at the height of movement restrictions enacted in response to the COVID-19 pandemic first wave. Phase Two: physical fitness of the original *N* = 62 children were determined directly pre- and post-isolation using an eight-component standardized fitness test battery and compared to *N* = 62 control children who were matched for age, sex, school region, and fitness centile scores.

**Results:** During lockdown (total duration: 63 days), moderate-to-vigorous physical activity (MVPA) decreased by ~46 min per day, screen time demonstrated a significant interaction effect, such that kids reported spending less recreational screen time on weekends during lockdown compared to no restriction, and sleep duration was consistently lower (95% CI: −104.1 to −45.5 min, *p* < 0.001). No interaction effect was present for direct fitness indicators, including: hand tapping (reaction time), standing broad jump, polygon backward obstacle course (coordination), sit-ups, stand-and-reach, bent-arm hang, 60-m, and 600-m run (*p* ≥ 0.05) although significant main effects are noted for both sexes.

**Conclusion:** Initial changes in 24-HMB did not translate to reductions in physical fitness *per se*, likely due to the high initial fitness levels of the children. Further work is needed to confirm whether longer or repeated movement restrictions exacerbate initial negative 24-HMB trends, especially for children who are less fit when restrictions are initiated, prolonged, or repeated.

## Introduction

Climate change is severely affecting all aspects of human life on Earth, in both direct (e.g. floods, drafts, wildfires) and indirect ways (e.g., ecosystem disruptions, increased air pollution, more aeroallergens) (1). The world is getting hotter (2), children are becoming less fit and more obese (3), and the likelihood of vector-borne diseases entering the human population is also increasing (1), rendering the possibility of future movement restrictions to novel disease outbreaks likely, itself creating a vicious-cycle of isolation, physical de-training (4), and increased risk for heat injury (5), especially for vulnerable populations like children. There needs to be greater priority given when considering children's health when creating public health policy (6), especially since the extraordinary impact the COVID-19 pandemic has had on human movement will likely not be a one-off situation.

From the earliest days of this COVID-19 pandemic, researchers have been sounding the alarm on how the negative impacts of isolation, confinement, and physical (in)activity will affect all persons (4), and children in particular (7). Problematically, most governments remain either unaware or unconcerned about the effects self-confinement have on the physical and mental health of its citizens (6). Jurak et al. have outlined the grave costs these restrictive measures have had on the physical fitness of children in Slovenia, noting that their research group has observed greatest decrease in child fitness in the >30 year history since systematic surveillance of child fitness began (6). To this point, there have been very few studies which have directly measured any indication of the deconditioning effect isolation has exerted on children. One recent study on *N* = 10 children reported that cardiorespiratory fitness (measured via VO_2peak_) was marginally lower in a “Pre” COVID-19 vs. “Post” COVID-19 group of otherwise healthy children (39.1 vs. 44.7 mL·kg^−1^·min^−1^, *p* = 0.031) (8). Similarly, a cohort study of *N* = 764 Austrian school children aged between 7 and 10 years taking place from September 2019 to September 2020, reported decreases in children's mean distance completed during a 6 min run from 917 ± 141 to 815 ± 134.3 m (*p* < 0.05) (9). There are no known current data assessing musculoskeletal decrements in children due to the COVID-19 pandemic. The effects of detraining on the physical performance of 7-year-olds has been previously investigated by Faigenbaum et al., who compared children's performance from two physical education classes, randomized into either an exercise (*n* = 20) or control group (*n* = 19) (10). Long jump, single-leg hop, curl-up, and balance were each assessed at baseline, after training, and after an 8-week detraining period. The authors reported significant group × time interactions after training for abdominal curls and single leg hop, whereas after detraining, the exercise group maintained their training-induced gains on curl-ups and single leg hop. The long jump regressed toward baseline for both groups. However, because children are in a rapid state of growth, detraining may induce fewer substantial, measurable deficits than those observed in adults. For example, researchers found that global responses to 4 weeks of detraining (after 8 weeks of leg press training) in 10–13-year-old, pre-peak-height-velocity stage boys, can persist over baseline measures for at least a month (11). The exercises they tested included assessments of boys' dominant and non-dominant limbs, unilateral one repetition maximum (RM) and 60% one RM, knee extension, knee flexion, handgrip maximal voluntary isometric contraction, and countermovement jumps. Importantly, Meylan et al. found that maturity can also modify the effects of strength training and detraining on performance, as observed from their study investigating 33 young men grouped based on the year(s) from/to age of predicted peak height velocity (12). The authors found that in the detraining period, the pre-peak-height group showed greatest loss of strength and power compared to children who were past their peak growth phase. They emphasized that maintenance programs are needed for most aspects of explosive performance, especially for less mature children. Thus, from the literature it appears that detraining effects in cardiorespiratory fitness and muscular strength are visible after 4–8 weeks of exercise cessation, and decreases may be more profound in less mature children.

Certainly, after 1 year of surveying the literature for changes in physical activity (PA) movement patterns during this pandemic, it is clear that there is no consensus on how best to promote and maintain adequate PA levels under movement restrictions (13, 14), that countries have varied and heterogeneous approaches to what sections of society are deemed “safe” or “unsafe,” “open” or “closed” (15) and that the vast majority of studies from individual countries report subjective measures of PA only (16–21), understandably due to an inability to safely (or legally) collect direct, objective, physical fitness data. Since Slovenia operates one of the largest continuous longitudinal databases of child fitness in the world (22), it was incumbent on the team to determine to what extent government-imposed restrictions of movement may have had on the fitness of children during the first wave of the COVID-19 pandemic. Thus, the purpose of this study was (1) to evaluate changes in children's 24-HMB using a robust repeated-measures design to inherently control for external factors like socioeconomic status, location, family dynamics, etc. and (2) to obtain direct physical fitness data to confirm whether the magnitude of (likely) changes in PA, especially moderate-to-vigorous physical activity (MVPA), were related to changes in child fitness, as soon as it was safe and legal to do so. It was hypothesized that after roughly 2 months of significant movement restrictions imposed by government policy during the first wave of the COVID-19 pandemic, there would be a decrease in weekday and weekend MVPA, an increase in recreational screen time, and a decrease in sleep duration reported. The secondary hypothesis was that any significant, negative trends observed in the 24-HMB of children during this first “lockdown” period would be significantly related to worsening physical fitness indices, directly measured within the first 4 weeks after lockdown restrictions were lifted.

## Materials and Methods

This study was approved by the Internal Review Board of the Faculty of Sport Ethics Commission, University of Ljubljana (No: 10/2018), following the Declaration of Helsinki for human studies. Permission to conduct the study was obtained from all teachers and principals at every participating school. Written, informed consent was obtained from all parents or guardians of the children, and positive assent was obtained verbally from all children prior to any data collection taking place. All participation was completely voluntary. Data were collected and analyzed anonymously.

### Study Design and Participants

Because of the sudden onset of the COVID-19 global pandemic, this study was initially conceived within the framework of an ongoing European project entitled “EUPASMOS”—The European Union Physical Activity and Sport Monitoring System project (Project no: 2017-3322/001/001). Briefly, the EUPASMOS project uses a multistage sampling design with balanced representation in terms of the geography, economic development, and rural-to-urban ratio of participants across its sample, with the smallest sampling unit defined as the family. The original study scope included 619 participants from nine schools. From this initial sample, 546 people were sampled across nine primary school districts, of which 30 participants were elderly (+65 years), 219 were children and adolescents (8–17 years), and 295 were adults. For the purposes of the present study, it was determined that EUPASMOS would provide an adequate existing infrastructure to query to what extent the Republic of Slovenia's pandemic restrictions may affect child health, therefore, participants (children) for this COVID-19 “sub-study” were recruited via e-mail by psychologists and kinesiologists from the Faculty of Sport, University of Ljubljana. The ethics IRB of the Faculty of Sport, University of Ljubljana approved the proposed COVID-19 follow-up study (No: 2020–274). A total of 154 children volunteered for the study in 2020. The final group consisted of sixty-two healthy children (*N* = 31 each boys and girls) who completed all portions of this COVID-19 direct fitness study.

**Figure 1:**
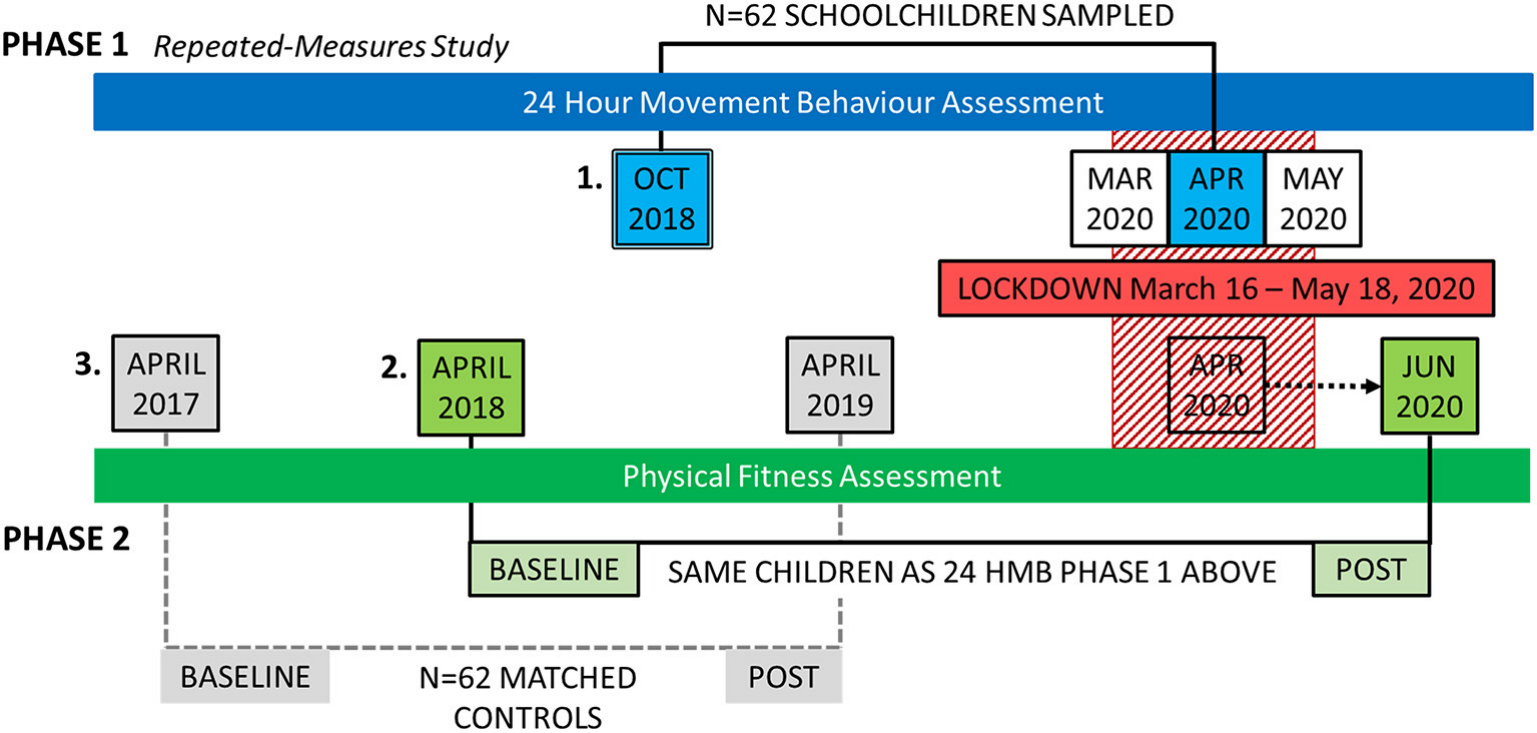
Study design and data collection timeline. (1) October 2018—As part of an ongoing European Project, 24-hour movement behavior (24-HMB) data from *N* = 62 Slovenian schoolchildren were used to compare to their own 24-HMB data at the height of movement restrictions in April 2020. (2) The physical fitness data of these children were extracted from the regular implementation of SLOfit, Slovenia's national fitness surveillance system for children for the time-point closest to October 2018 (which was April 2018). This fitness data under normal living conditions were then compared with children's fitness data obtained immediately after movement and school restrictions were lifted (June 2020). (3) Finally, to determine whether the differences observed in child fitness between sampling time points was considered normal, data were interrogated for *N* = 64 matched-control children from the SLOfit database. The control children were matched for age, sex, school region, and fitness centile value to the original children from the October 2018 study. To make sure the 2-year time interval between testing was respected (but to avoid the 2020 data collection pandemic year), these children's fitness data were extracted for SLOfit test years April 2017 and April 2019, respectively.

The study design and timeline for data collection are communicated in Figure 1, using a two-phase approach (Phase One: 24-HMB assessment, and Phase Two: Physical fitness assessment). Briefly, Phase One of the study was completed first, and consisted of compiling data from the *N* = 62 school children who completed their self-reported questionnaire during the height of the pandemic lockdown (April 2020). This data was compared with their previous responses submitted pre-pandemic (October 2018), in a repeated-measures fashion. In Phase Two, a repeated-measures, matched-control study was designed to utilize Slovenia's longitudinal child fitness surveillance database “SLOfit” (22), where fitness data of the children who participated in Phase One were extracted from two time-points, immediately post COVID-19 restriction in June 2020, and pre-pandemic in April 2018.

### Phase One: Data Collection Timeline for 24-HMB Assessment

Data collection was performed in the months of October and November 2018, and in April 2020 during the height of the initial, 2 month lockdown required by the government of the Republic of Slovenia to counteract the first wave of COVID-19 infection. The children were in a “lockdown” scenario for 63 days (from 16 March 2020) during which most social services were suspended. Children (and their parents/guardians) were first contacted for their participation 24 April 2020; a first reminder was sent shortly thereafter (11 May 2020), and a second reminder 2 weeks subsequently (18 May 2020) for any remaining respondents. Children remained out of the Slovenian school system until 18 May 2020.

### Phase One: Assessment of Physical Activity, Screen Time, and Sleep

In October 2018, children completed a series of online questionnaires querying their PA, screen time, and sleep habits as part of the EUPASMOS data collection. Testing took place on a weekday in the afternoon. The same children then completed the identical questions in April 2020, using an online platform (1KA, University of Ljubljana, Faculty of Social Sciences). They were familiarized with using this online system previously. Physical activity was assessed with School Health Action, Planning, and Evaluation System (SHAPES) (23), which was back translated from English to Slovenian by four native Slovenian speakers, following World Health Organization (WHO) recommendations for translation and adaptation of instruments (24). The SHAPES questionnaire has adequate reliability and validity (25). Moderate-to-vigorous physical activity was calculated based on daily self-reported moderate and vigorous minutes (moderate + vigorous minutes). Screen time was determined by summing the variables used to assess total screen time (e.g., time spent watching television, watching videos on computer or DVD, using cell phone, playing videogames, browsing on the internet). The Pediatric Daytime Sleepiness Scale was used to determine total sleep time (26). Variables (MVPA, sedentary time, sleep) are expressed in minutes of activity, calculated separately for weekdays and weekends, and summed for a total minutes per week value.

### Phase Two: Data Collection for Physical Fitness Assessment

For over the past 30 years, the vast majority of Slovenian schoolchildren (aged 6–19 years) participate in a nation-wide, school-based physical fitness surveillance programme, “SLOfit” which consists of eight fitness tests and three anthropometric tests within its test battery (22). It is performed every April in every public school across Slovenia. The system is described in detail elsewhere (22) but briefly, in addition to measuring height, mass, and triceps thickness, the fitness test battery consists of completing eight tasks: hand tapping (reaction time), standing broad jump (lower-body power), polygon backward obstacle course (coordination), sit-ups (muscular endurance), stand-and-reach (flexibility), bent-arm hang (upper-body endurance), 60-m run (sprint), and 600-m run (endurance). More than 95% of all Slovenian schoolchildren attend public school; there are only three “private” schools in the country, and thus, the database accurately reflects the entire pediatric population of Slovenia. In addition to providing students and their parents feedback on their child's physical and motor development and associated health-risk(s), this system allows teachers and researchers access to high-quality, standardized data on physical fitness, which is then used to directly inform public policy.

After Slovenia declared an “end” to the first wave of the epidemic on 15 May 2020, certain physical distancing measures were relaxed, allowing students to gradually return to school. Due to school closures, the regular annual SLOfit testing could not be carried out in April, but there were 20,000 students aged 6–14 years who completed fitness measurements from mid-May to June, demonstrating some exceptional school organization and commitment on the part of the individual physical education teachers. Thus, the children comprising the present sample were evaluated during their regular SLOfit testing in April 2018, and then again in June 2020, approximately 24–26 months after the first measurements were completed, and between 1 and 4 weeks after schools and other societal activities were re-opened.

### Phase Two: Selection of Matched Controls for Physical Fitness Assessment

To assess whether the progression of individual fitness components comprising the SLOfit test battery were progressing at a normal rate based on the children's age, researchers decided to add a “Phase Two” matched-control aspect to the present study. A sub-group of *N* = 62 completely unique children were interrogated from the SLOfit database, matched for: age, sex, school region, and centile performances for each fitness variable. To ensure completely different children were selected (and to avoid any unknown effect of pandemic-regulations on other aspects of the children's lives), kids were selected from the base year April 2017. Data were then extracted for those same children in April 2019, the last year when “normal” fitness testing surveillance took place.

### Statistical Analysis

A repeated measures analysis of variance with Bonferroni correction was used to determine any significant differences between groups. The determination of movement behaviors analysis consisted of two within-subjects factors (year: 2018 and 2020, week: weekday, weekend) and one between subjects factor (sex: male, female), with alpha levels set at *p* < 0.05 level of significance. A power analysis was performed for the sample (*n* = 62), separated by sex (*n* = 31), which yielded adequate power for this investigation (0.932). Physical fitness variables were analyzed using a repeated measures analysis of variance with Bonferroni correction with one within-subjects factor (year: baseline, post) and one between-subjects factor (study: experimental group, matched controls). Fitness data were analyzed separately by sex since these comparisons were not central to the principle questions being investigated. Pairwise, two-tail bivariate correlations were run on the change scores from 2018 to 2020 for MVPA, screen time and sleep duration. Data are presented as means and standard deviations, with 95% confidence intervals, *t*-values, *F*-ratios, and effect size (Cohen's *d* and η^2^) where appropriate. Normal distribution was checked with Q–Q plots and the homogeneity of variances with Levene's test. Paired *t*-tests or independent *t*-tests were used to determine group effects when a significant interaction effect was present. If there were no significant differences between the weekday/weekend variables, or between sexes, data were collapsed between-group, and group data are reported in-text. All statistical analyses were calculated using SPSS 27.0 (IBM Inc., Chicago, USA).

## Results

### Sample Characteristics

Participant characteristics are detailed in Table 1. There were no significant differences between the children originally recruited in Phase One of the project and their Phase Two matched controls later retrieved from the SLOfit fitness surveillance database.

**Table 1:** Physical characteristics of the children initially recruited to participate in the study, and the matched controls extracted for Phase 2, physical fitness assessment.

**Variable**		**2018 Original sample**	**Matched controls**	***p*-Values**
Age (years)	Boys	11.1 ± 1.5	11.1 ± 1.5	0.990
		(7.0–14.0)	(7.0–14.0)	
	Girls	12.0 ± 1.5	11.9 ± 1.5	0.711
		(10.0–15.0)	(10.0–15.0)	
	All	11.6 ± 1.5	11.5 ± 1.5	–
		(7.0–15.0)	(7.0–15.0)	
Height (m)	Boys	1.51 ± 0.11	1.51 ± 0.11	0.978
		(1.31–1.73)	(1.31–1.73)	
	Girls	1.55 ± 0.10	1.54 ± 0.10	0.860
		(1.29–1.69)	(1.31–1.69)	
	All	1.53 ± 0.11	1.52 ± 0.10	–
		(1.29–1.73)	(1.31–1.73)	
Mass (kg)	Boys	41.7 ± 10.9	42.1 ± 11.2	0.891
		(24.9–75.4)	(26.7–69.3)	
	Girls	45.8 ± 10.6	44.9 ± 11.2	0.749
		(25.0–76.9)	(27.3–71.1)	
	All	43.7 ± 10.9	43.4 ± 11.2	–
		(24.9 ± 76.9)	(26.7–71.1)	
BMI	Boys	18.1 ± 2.5	18.2 ± 2.8	0.842
		(14.6–25.5)	(14.3–26.8)	
	Girls	18.9 ± 2.7	18.6 ± 3.2	0.743
		(14.9–27.2)	(14.4–26.3)	
	All	18.5 ± 2.6	18.4 ± 3.0	–
		(14.6–27.2)	(14.3–26.8)	
Triceps thickness (mm)	Boys	12.0 ± 4.0	10.4 ± 3.9	0.143
		(7.0–22.0)	(4.0–29.0)	
	Girls	13.0 ± 4.7	13.0 ± 4.4	0.976
		(7.0–25.0)	(8.0–24.0)	
	All	12.5 ± 4.3	11.7 ± 4.3	–
		(7.0–25.0)	(4.0–24.0)	

*Data are means ± standard deviation with ranges (min-max) in brackets. There were no significant differences between the 2018 originally-sampled children and their matched controls (within-sex) extracted from the SLOfit database*.

### Phase One: Effects of COVID-19 Lockdown Measures on 24-HMB in Children

**Figure 2:**
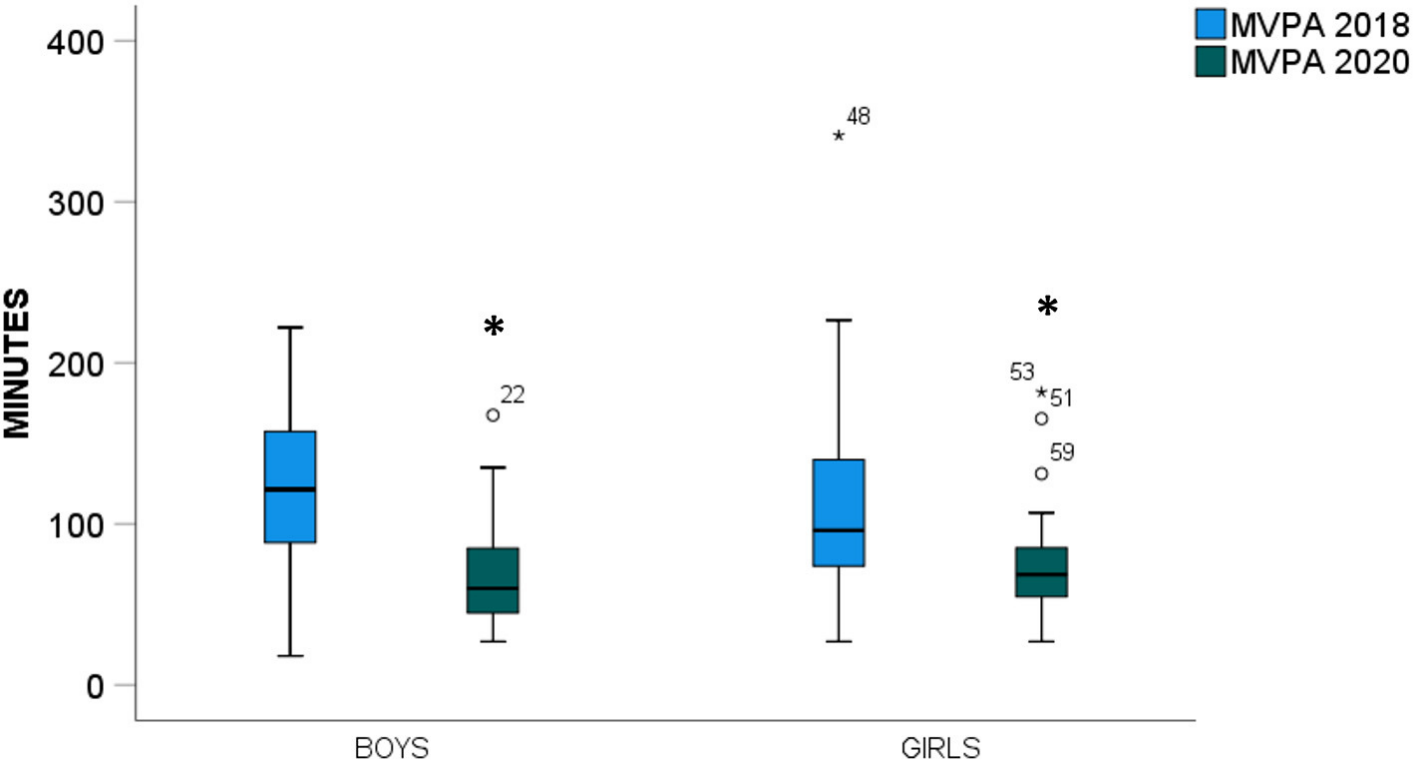
Moderate-to-vigorous physical activity (MVPA) for boys and girls from October 2018 (blue) and April 2020, when COVID-19 movement restriction policies were in effect (green). *Indicates a significant main effect between time-points (*p* < 0.05).

**Figure 3:**
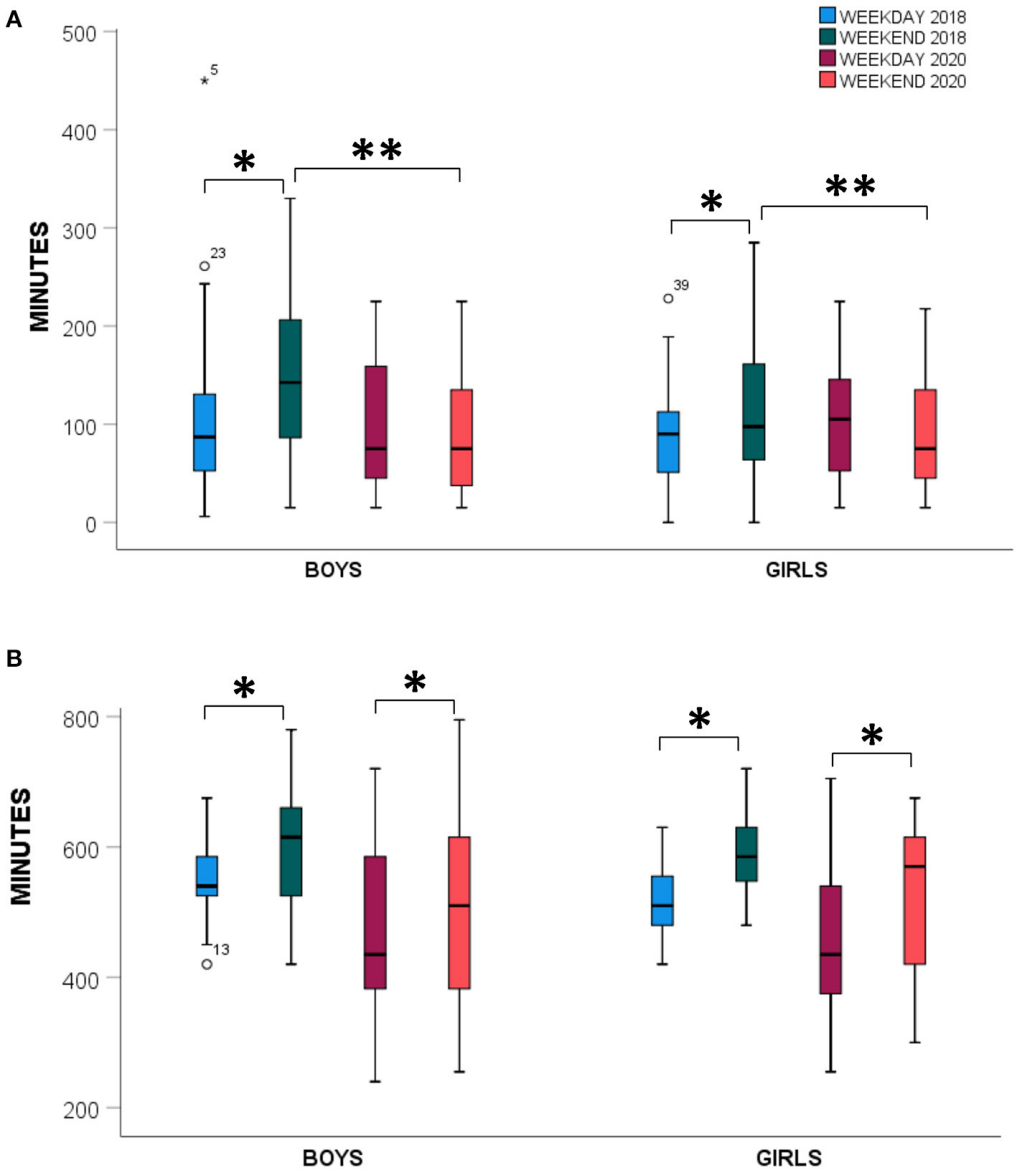
**(A)** Self-reported cumulative screen time (min) for boys and girls during October 2018 weekdays (blue), October 2018 weekends (green), and during the COVID-19 lockdown weekdays (red) and weekends (orange). **(B)** Sleep duration (min) using identical coloring to **(A)**. *Indicates a significant main effect between time-points, **Weekend data are significantly different between 2018 and 2020 (*p* < 0.05).

Moderate-to-vigorous physical activity was significantly affected by the COVID-19 lockdown measures such that self-reported MVPA minutes decreased substantially, by ~46 min per day (95% CI: −32.1 to −59.5 min, *p* < 0.001, *F* = 44.6, Table 2). These reductions were independent of interaction effects by sex (*p* = 0.184, *F* = 1.804) or weekday/weekend values (*p* = 0.322, *F* = 0.996). Between-subject effect of sex were also not significant (*p* = 0.758, *F* = 0.095, Figure 2). Screen time demonstrated a significant two-way interaction between the year of measurement and sex (*p* = 0.047) such that during regular societal functioning, screen time was ~34% higher on weekends than weekdays (Boys: 147 ± 75 vs. 106 ± 90 min, Girls: 115 ± 69 vs. 90 ± 53 min, respectively). This was not the case during COVID-19 lockdown, when the trend was reversed, and children reported spending ~94 min less screen time on weekends than under normal circumstances (Boys: 93 ± 61 vs. 97 ± 69 min, Girls: 95 ± 61 vs. 111 ± 64 min, Figure 3A). Sleep duration was higher on weekends than weekdays, regardless of during COVID-19 lockdown or no restrictions (95% CI: 33.6 to 82.1 min, *p* < 0.001). Sleep duration was also consistently lower during the COVID-19 lockdown than when no restrictions were in place (95% CI: −104.1 to −45.5 min, *p* < 0.001). There were no interactions observed for sleep duration and sex (*p* = 0.350, *F* = 0.886, Figure 3B).

**Table 2:** Phase 1 repeated-measures differences in moderate-to-vigorous physical activity (MVPA), screen time minutes and nocturnal sleep duration sampled in October 2018 and April 2020, stratified between boys and girls.

	**2018 No restriction**			**2020 Lockdown**		
	**Weekday**	**Weekend**	**Combined**	**Weekday**	**Weekend**	**Combined**
**MVPA**						
Boys	130.2 ± 45.5	118.1 ± 65.9	124.2 ± 49.0	69.6 ± 35.0	68.7 ± 39.8	69.1 ± 35.7
Girls	117.2 ± 68.2	106.7 ± 82.7	111.9 ± 62.5	72.4 ± 32.4	78.3 ± 43.0	75.4 ± 34.7
All	123.7 ± 59.9	112.4 ± 74.4	118.1 ± 56.0	71.0 ± 33.5	73.5 ± 41.4	72.2 ± 35.0^*^
**SCREEN**						
Boys	105.7 ± 89.8	146.9 ± 74.8	126.3 ± 76.0	97.1 ± 68.6	92.7 ± 61.3	94.9 ± 52.9
Girls	89.6 ± 53.5	114.7 ± 68.9	102.1 ± 54.3	110.8 ± 63.5	95.1 ± 61.3	102.9 ± 50.9
All	97.6 ± 73.8^a^	130.8 ± 73.1^a,c^	114.2 ± 66.6	103.9 ± 65.9	93.9 ± 60.8^c^	98.9 ± 51.7
**SLEEP**						
Boys	549.2 ± 57.3	600.5 ± 88.0	574.8 ± 52.3	475.6 ± 141.8	497.0 ± 142.3	486.3 ± 108.3
Girls	516.8 ± 52.8	587.4 ± 59.8	552.1 ± 45.8	447.1 ± 118.5	535.2 ± 135.8	491.1 ± 99.2
All	533.0 ± 57.0^a^	594.0 ± 74.9^a^	563.5 ± 50.1	461.4 ± 130.9^b^	516.0 ± 139.3^b^	488.7 ± 103.0^*^

*Data presented are means ± standard deviations, expressed in mean minutes per day. The heading term “Combined” refers to the average number of min per day for the entire week. Superscript matching letters denote significant year and weekday/weekend interaction for that variable and timepoint (p < 0.05)*.

* *Significant main effect difference from 2018 for that variable (p < 0.05)*.

Bivariate correlations were run on change scores of the dependent measures between sampling years (Table 3). Relationships were generally non-significant, although differences in weekday/weekend MVPA (*p* = 0.042) and weekday/weekend SLEEP (*p* = 0.010) were (unsurprisingly) related. Centile values for the eight standardized fitness indicators for children initially recruited to participate in the project and the matched controls extracted for Phase 2 physical fitness assessment portion of the project are communicated in Table 4. Both girls and boys in the current study were within the top third for their age and sex compared to the general population within a given fitness indicator.

**Table 3:** Bivariate correlations to difference scores (2018 no restriction minus 2020 lockdown values) in 24-HMB dependent variables, differentiated by weekday and weekend.

		**MVPA**		**SCREEN**		**SLEEP**	
		**Weekday**	**Weekend**	**Weekday**	**Weekend**	**Weekday**	**Weekend**
MVPA weekday	ρ	1	0.259	−0.071	0.179	−0.039	−0.181
	*p*-Value		0.042^*^	0.581	0.163	0.766	0.160
MVPA weekend	ρ	0.259	1	−0.080	−0.098	0.079	0.097
	*p*-Value	0.042^*^		0.537	0.449	0.542	0.453
SCREEN weekday	ρ	−0.071	−0.080	1	−0.323	0.036	−0.059
	*p*-Value	0.581	0.537		0.010^*^	0.783	0.649
SCREEN weekend	ρ	0.179	−0.098	−0.323	1	0.220	0.149
	*p*-Value	0.163	0.449	0.010^*^		0.085	0.248
SLEEP weekday	ρ	−0.039	0.079	0.036	0.220	1	0.159
	*p*-Value	0.766	0.542	0.783	0.085		0.217
SLEEP weekend	ρ	−0.181	0.097	−0.059	0.149	0.159	1
	*p*-Value	0.160	0.453	0.649	0.248	0.217	

* *Significant correlation p < 0.05*.

**Table 4:** Centile values for the eight standardized fitness indicators for children initially recruited to participate in the project, and the matched controls extracted for Phase 2 physical fitness assessment portion of the project.

**Fitness indicators**		**2018 Original sample**	**Matched controls**	***p*-Values**
Tapping	Boys	63.7 ± 27.4	63.0 ± 26.1	0.916
(reaction time)	Girls	61.9 ± 24.7	61.7 ± 26.9	0.970
Standing Broad Jump	Boys	63.1 ± 29.0	64.9 ± 29.2	0.812
(lower-body power)	Girls	61.8 ± 25.8	67.2 ± 22.5	0.419
Polygon Backward	Boys	84.0 ± 16.6	63.2 ± 26.7	0.001
(coordination)	Girls	76.4 ± 26.0	72.1 ± 22.8	0.524
Sit-up	Boys	77.6 ± 20.7	61.5 ± 28.5	0.018
(muscular endurance)	Girls	80.0 ± 19.7	74.1 ± 23.5	0.318
Stand-and-reach	Boys	63.6 ± 24.7	59.0 ± 33.6	0.557
(flexibility)	Girls	69.3 ± 26.4	52.0 ± 31.0	0.032
Bent-arm hang	Boys	72.8 ± 22.5	71.4 ± 24.3	0.826
(upper-body endurance)	Girls	63.4 ± 29.1	72.0 ± 30.6	0.302
60-m run	Boys	67.6 ± 29.9	65.5 ± 25.5	0.779
(sprint)	Girls	70.4 ± 27.3	71.2 ± 21.9	0.900
600-m run	Boys	64.7 ± 29.4	64.5 ± 25.2	0.969
(endurance)	Girls	70.6 ± 25.1	75.9 ± 23.6	0.439
Fitness index^a^	Boys	79.0 ± 24.5	72.8 ± 25.2	0.358
	Girls	77.1 ± 26.6	76.0 ± 23.8	0.877

*Values are means ± standard deviations*.

a *The Fitness Index is an amalgamation of all eight fitness indicators. It is used primarily as a surveillance tool within Slovenia to compare children's fitness over the >33 year history of the SLOfit programme*.

### Phase Two: Physical Fitness Indices

**Figure 4:**
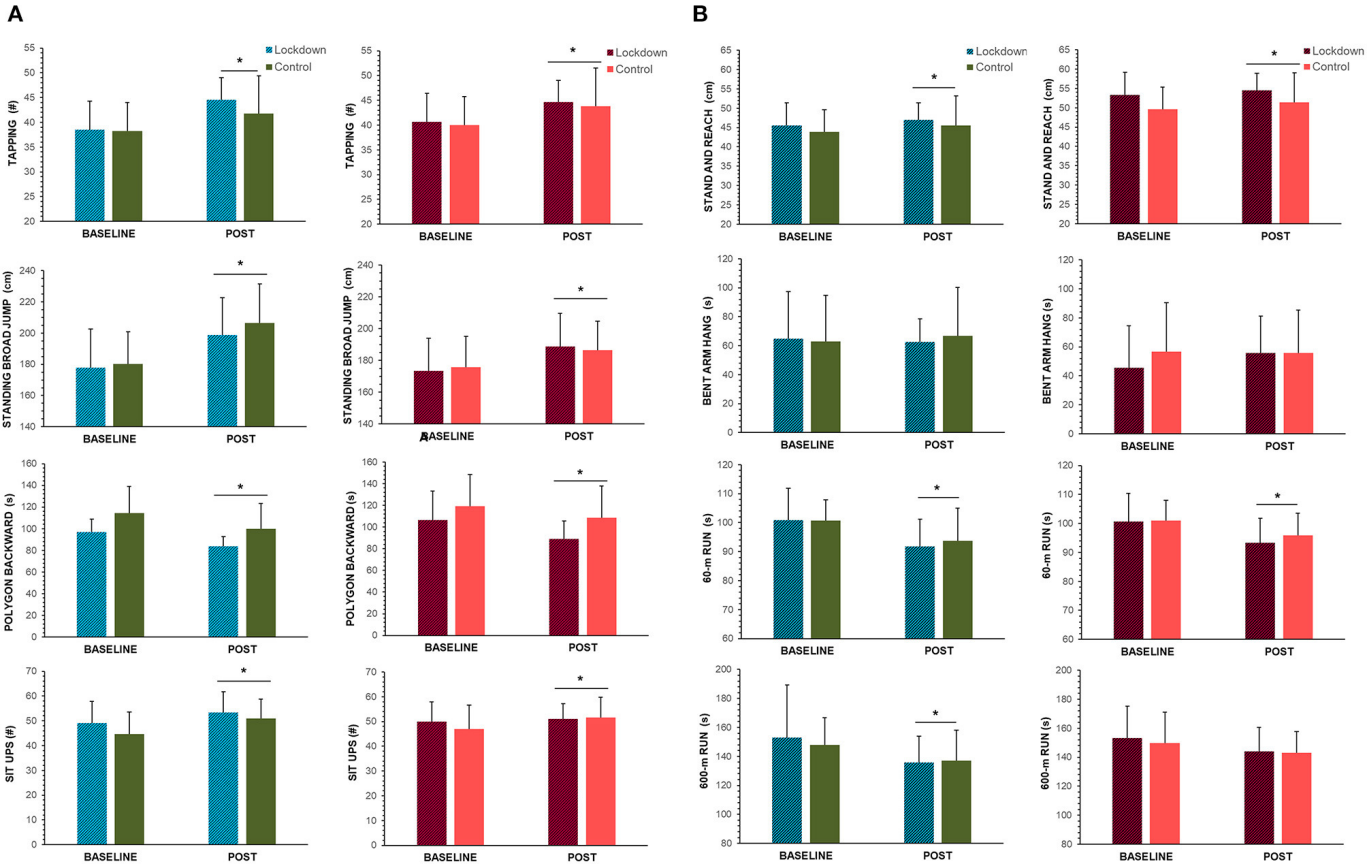
Fitness data collected for **(A)** the first four and **(B)** last four fitness indices during the regular implementation of SLOfit, Slovenia's national fitness surveillance system for children. On the x-axis, “BASELINE” refers to data collected April 2018 for “lockdown” group, and April 2017 for matched controls (See Figure 1 for timeline of data collection and full study description). “POST” measures refer to fitness testing conducted immediately post-lockdown (June 2020) for the “lockdown group,” and April 2019 for matched controls. Mean fitness data are depicted for boys (blue and green bars) girls (red and orange bars), with standard deviation error bars. *Indicates a significant time main effect between baseline and post measurements (*p* < 0.05).

There were no significant interactions between experimental group and matched controls for any of the eight fitness tests comprising the SLOfit standardized test battery (Figures 4A,B) for either girls or boys (*p* > 0.05), beyond the significant time main effect naturally expected between surveillance measurements (i.e., any differences observed between the first and second fitness testing, respectively). Main effect comparisons between Baseline and Post measures included: plate tapping (boys: from 39 ± 5 to 43 ± 7, girls: 39 ± 4 to 44 ± 5 taps, *p* < 0.001), standing broad jump (boys: from 180 ± 20 to 203 ± 25, girls: 172 ± 16 to 188 ± 19 cm, *p* < 0.001), polygon backwards (boys: 108 ± 25 to 93 ± 21, girls: 110 ± 26 to 102 ± 27, *p* < 0.001), sit-ups (boys: 47 ± 8 to 52 ± 8, girls: 47 ± 8 to 51 ± 8 count, *p* < 0.001), sit-and-reach (boys: 45 ± 7 to 46 ± 8, girls: 49 ± 8 to 53 ± 7 sit-ups, *p* < 0.001), 60 m run (boys: 100 ± 7 to 93 ± 11, girls: 102 ± 7 to 95 ± 8 s, *p* < 0.001), and 600 m run (boys: 145 ± 13 to 137 to 20 s, *p* < 0.024). There were no differences in 600 m run times for girls (147 ± 13 to 143 ± 15 seconds, *p* = 0.121). Bent-arm hang time was not different for either sex (boys: 62 ± 29 to 65 ± 28, girls: 57 ± 31 to 57 ± 28 s, *p* = 0.566). Since there were no significant interaction effects present between test groups, no further analyses were performed between fitness indices and 24-HMB data.

## Discussion

This study confirmed that a 2-month “lockdown” style reduction in enforced physical movement restriction adversely affects MVPA, independent of time of week (i.e., both weekdays and weekends) for both boys and girls. Recreational screen time on weekends was reduced compared to pre-pandemic data, and sleep duration demonstrated large variability during both weekdays and weekends. For this sample of children, there were no changes in PF scores compared to anticipated changes observed for the same duration in children who did not experience lockdown measures. Therefore, although self-reported MVPA was affected by nationally imposed movement restrictions, these measures did not translate into consistent, specific, fitness-related decreases over the 63-day restriction period, within this sample at least.

### Effects of Movement Restriction Policies on 24-HMB in Children

The 24-HMB of children worldwide has been dramatically altered throughout the COVID-19 pandemic (21). Although health benefits for attaining adequate levels of MVPA and sleep, together with reducing sedentary behaviors (like screen time) are well-established and evidence-based (27), negative trends in all three movement behaviors have been observed in children throughout the first-wave of the COVID-19 pandemic worldwide. Indeed, children's MVPA tended to decrease, screen time increase, and sleeping patterns worsen independent of geographic location (18, 28–30). The case for Slovenian schoolchildren appears to be no different. However, even though the MVPA of schoolchildren in the current study was reduced (by more than 46 min per day), they were still generally meeting WHO PA guidelines (31), whereas in other countries, this was not always the case (17, 18, 30, 32). Discrepancies between countries may be due to the aggressive public health policies Slovenian sport experts undertook early in the first wave. Countermeasures to reduce physical inactivity in Slovenia have been detailed elsewhere (7), but briefly, experts prepared and published PA guidelines for public use within the first 5 days' of WHO declaring COVID-19 a pandemic (33) encouraging engagement in outdoor physical activities, exercise at home, and national television broadcast stations televised 1 h of physical exercises led by physical education teachers on public television and live-streamed over social media platforms daily. Nevertheless, although children were meeting general PA guidelines in terms of total minutes moving per day, and Slovenian citizens were being encouraged to remain physically active, it appears from the current study that children's PA intensity was compromised compared to the pre COVID-19 era. When participating in organized sport and in physical education lessons, children's PA is more intense; one study has found that boys in school situations get most of their daily MVPA in this way (34). As Schmidt et al. noted in a German sample of children, habitual PA in fact increased significantly during their lockdown, but the kids were lacking guided, organized MVPA (28). Contrary to other countries' screen time data, Slovene children reported decreased levels of recreational screen time, especially during the weekend. We must emphasize that the SHAPES questionnaire is designed to measure *leisure* screen time, with questions focusing on watching TV or movies and spending time on the computer for fun (playing games, browsing the web, etc.). Therefore, despite decreases in leisure screen time, overall screen time likely increased dramatically since children were schooling online and spending most of their days indoors. Screen time guidelines for children suggest spending <60 min per day across all mediums, but of course all children were vastly exceeding this recommendation in the COVID-19 era due to completing demanding home-schooling and remote learning activities. Since methodological limitations of the questionnaire address leisure screen time specifically, we can only assume that Slovene children's total screen time is far higher during the COVID-19 lockdown; however, the reported decreases in leisure screen time is nevertheless positive, suggesting that children opted to spend their spare time doing other activities.

Overall sleep duration decreased significantly during lockdown, with many Slovene children no longer meeting optimal sleep guidelines (35) of 9–11 h during weekdays. Other studies investigating child sleep habits during the COVID-19 outbreak are variable, with some reporting increases in sleep time (17, 18, 30), possibly due to more flexible schedules and absence of school commuting (21). The case in Slovenia is different since children reported more variable sleep patterns during lockdown. These trends could be connected to the late use of mobile devices or additional screen time at bed, which is known to have a negative effect on sleep quality (36). And indeed, the present study determined there were modest connections between weekday/weekend sleep and screen patterns in this cohort (Table 3). Since all three 24-HMB affect one another, spending more time engaging in one activity will certainly affect the other movement behaviors. Therefore, it is critical for there to be an established and maintained home routine, preferably one similar to school days, which encompasses strategies for all three healthy behaviors to maintain children's (and parent's!) mental and physical health, especially during times of disruption and heightened stress.

### MVPA and the Importance of Maintaining Physical Activity Intensity

Attaining sufficient PA intensity in addition to the overall PA minutes is crucial for maintaining physical fitness, but the sudden onset of the COVID-19 global pandemic and its subsequent physical distancing/lockdown regulations forced people (and specifically, children) to “stay at home” with the consequence that children spent far less time engaging in PA, and far more time being sedentary, usually indoors. Higher levels of MVPA in children are associated with lower adiposity, lower cardiometabolic risk factors, and better cognitive function (37–39). In terms of the COVID-19 pandemic, the total direct costs of secondary care during the first wave of COVID-19 (in Europe) have been estimated to be ~13.9 billion euros (16.97 billion USD), in which 76% of these costs were accounted for treating people (adults) who present as overweight or obese (40). Dumuid et al. have suggested that *avoiding declines* in MVPA is even more important for promoting health outcomes than efforts to increase MVPA *per se*, especially among inactive children (41). Prolonged confinement negatively affects mental health, cardiovascular and metabolic function, and sleep (42–44), and as the MVPA of Slovenian school children decreases, there may yet be direct, lasting, negative effects on aerobic function, musculoskeletal fitness, and mental health (42), especially since overall physical fitness is so closely connected to MVPA. Considering the prolonged, repeated periods of increased sedentarism during regional lockdowns globally, children are missing out on vital opportunities to engage in organized, quality, high-intensity PA that make up the basis for maintaining fitness, especially for those of lower fitness states. It is for this reason that the current updated WHO guidelines on PA and sedentary behavior (31) may not go far enough to avoid declines in MVPA, especially when children are experiencing repeated lockdown events, and specifically for less active children.

### No Interaction Effect of First-Wave Movement Restrictions on Physical Fitness Parameters

The current investigation did not observe significant interactions between fitness variables in the lockdown sample vs. the matched controls. The lack of significance can be largely attributed to four factors, (i) Slovenian environmental conditions, (ii) baseline fitness of the children, (iii) Slovenian countermeasure actions to the first-wave lockdown, (iv): the post-testing window. The first wave lockdown of the Slovenian society occurred during an unseasonably warm and dry spring, during which much of the population was encouraged to remain “socially-distanced” but were permitted to venture outdoors. Data interrogated from the Google Mobility reports found that the Slovenian population frequented parks, green spaces, and marinas more than pre-lockdown periods. These data are reported elsewhere (7), but the “take home message” is that the Slovenian population went outside a lot, and this aligns with the data herein that report children being more active on weekends. Likely, these outings were leisurely, hence the intensity of PA was not high, but indeed, Slovenia's first lockdown was not as “harsh” as other neighbor countries (15), indicating it was possible to get outside and enjoy nature. (b) Reductions in MVPA likely did not affect fitness indices because the children included in the sample came from relatively high fitness centiles compared to children nation-wide, who themselves have cardiorespiratory fitness levels corresponding to healthy ranges (45) compared to children in other countries. When using the SLOfit database to find matched controls for Phase 2 of this study, it became apparent that these children were at less risk for increased sedentary behaviors or poor fitness outcomes. This may be (at least, in part) because (c) Slovenia took swift action to communicate countermeasures to physical inactivity to the physical education teachers and general population during the first lockdown. These countermeasures are described elsewhere (7, 33), but they included not just outreach on social media platforms, but engaging trained pedagogical physical education specialists to reach out to children as they continued their remote schooling (33). Finally, (d) there were up to 4 weeks between the end of government enforced “lockdown” measures and direct fitness testing taking place in schools. During this time, some fitness indices may have returned to normal for the children, which itself is an encouraging finding. It is unclear whether longer lockdowns, repeated lockdowns, and/or seasonal variations may play a role in future fitness trends in children worldwide.

## Study Considerations

This is the first study to investigate 24-HMB and incorporate direct measurement of child fitness both before and immediately after physical distancing measures were placed due to the COVID-19 pandemic. Data were collected in a repeated-measures fashion on *N* = 62 schoolchildren, which the authors concede is a low sample size compared to literature investigating PA in children. The sample size is much higher than many other studies with direct physical measurements, including the only known source of direct cardiorespiratory fitness measurement of healthy children (8) in COVID-19 pandemic times. The reliability of the SHAPES questionnaire is adequate for this sample; unfortunately, direct measurement of PA (e.g., accelerometers) were not possible within the scope of this study. Thus, PA was assessed indirectly, during the “shoulder” seasons of fall and spring. Although temperatures in Slovenia are similar during these times of year, the authors acknowledge there may be slight differences in family activity patterns, especially when considering outdoor exposure or active play experiences of the children. This study recruited peripubertal boys and girls, an age at which maturation, puberty, and growth rate can each significantly affect fitness, physical literacy, and overall PA, thus representing a confounding factor in the evaluation of isolation/inactivity on one's fitness. This group was selected because the authors believed this age may have been at greater risk for the dual-pronged issue of continuing to be dependent on the family unit for exposure to quality PA (i.e., still not fully independent), yet also burdened with increased sedentary activity, especially from more remote work (e.g., online learning). Because this study was not able to determine biological age independent of chronological age, it was designed using matched controls to consider indirectly the effect of puberty on a given sample. Thus, improvements seen in some fitness variables might be attributed more to one's natural timeline of maturation, than any reductions seen in MVPA *per se* (i.e., the isolation stimulus was not great enough to overcome the natural increases coinciding with growth and maturation of the child). Finally, as mentioned in the discussion above, the children in this sample were comprised mainly of a very fit and active participants. Whether these results are nationally-representative is not known, but preliminary fitness testing from a much larger sample suggests this is not the case, and caution should be practiced when interpreting the results of this study. Indeed, these data represent a “best case scenario” regarding the effects of physical distancing and isolation on (relatively) highly fit children.

## Conclusions

Children reported completing less MVPA per day at the height of movement restrictions during the first wave of the COVID-19 pandemic. Recreational screen time on weekends was lower during the COVID-19 pandemic compared to no restrictions, likely due to alterations in family movement patterns. Children's sleep duration was more variable under movement restriction than normal. These changes in 24-HMB were not reflected in indices of physical fitness, likely due to the relatively “short” duration of movement restrictions in place for the first wave of the COVID-19 pandemic, and the high initial fitness levels of the children. Further work is needed to confirm whether longer or repeated movement restriction durations would exacerbate the initial negative 24-HMB trends observed, especially for children who are less fit when restrictions are first initiated.

## Data Availability Statement

The raw data supporting the conclusions of this article will be made available by the authors, without undue reservation.

## Ethics Statement

The studies involving human participants were reviewed and approved by Internal Review Board of the Faculty of Sport Ethics Commission, University of Ljubljana (No: 10/2018). Written informed consent to participate in this study was provided by the participants' legal guardian/next of kin.

## Author Contributions

SM: conceptualization, formal analysis, visualization, writing—original draft, review and editing, and approving final submission. VS: investigation, data curation, formal analysis, writing—review and editing, and approving final submission. KM: investigation, data curation, writing—review and editing, and approving final submission. GS and GJ: project administration, conceptualization, investigation, resources, writing—review and editing, and approving final submission. All authors contributed to the article and approved the submitted version.

## Funding

Limited non-specific funding was provided by the Slovenian National Research Agency (P5-0142 Bio-Psycho-Social Context of Kinesiology). The Phase 1 portion of the research design was partially co-funded by the Erasmus + Programme of the European Union within the project title EUPASMOS (No. 590662-EPP-1-2017-1-PT-SPO-SCP).

## Conflict of Interest

The authors declare that the research was conducted in the absence of any commercial or financial relationships that could be construed as a potential conflict of interest.

## Publisher's Note

All claims expressed in this article are solely those of the authors and do not necessarily represent those of their affiliated organizations, or those of the publisher, the editors and the reviewers. Any product that may be evaluated in this article, or claim that may be made by its manufacturer, is not guaranteed or endorsed by the publisher.
